# Statistical Inquiry into the Present State of the Medical Charities of Ireland: With Suggestions for a Medical Poor-Law, by Which They May Be Rendered Much More Extensively Efficient

**Published:** 1836-07

**Authors:** 


Art. IV.—*Statistical Inquiry into the present State of the Medical Charities of Ireland: with Suggestions for a Medical Poor-Law, by which they may be rendered much more extensively efficient.* By Denis Phelan, Surgeon to the Co. Tipperary Gaol.—*Dublin,* 1835. 8vo. pp. 325.

The objects of the medical institutions of a country are the public good, which they either promote directly, by healing the sick and restoring them to their functions in society, or more remotely, by extending and advancing science. Whether, with all the means at their disposal, they answer the great purposes of their existence, is a question equally deserving the attention of an enlightened government and of the medical profession; particularly when any extension of their application or any organic change in their regulation is contemplated. To solve this question,—to show the wants of the sick poor in Ireland,—the extent, the abuses, the inefficiency of her medical charities, and the means of remedy,—the Statistical Inquiry before us is a valuable document: it shows, on the one hand, the frequent sickness and wretchedness of an impoverished people, arrested at a stage of half-civilization; on the other, the institutions intended for their amelioration often wasting and misusing the stock of good at their disposal. Notwithstanding, however, the gloomy picture of the Irish poor drawn by Mr. Phelan, and fully borne out by the Irish Poor-law Commissioners, their present condition is really better than that of their forefathers, and they are exposed to no more privations, famines, or diseases, than other families of mankind in the same stage of civilization. But why is Ireland left so far behind England? Why does the general mass of her people still grovel in the wretchedness of the feudal ages of Europe? The profuseness of riches, the refined civilization, the princely mansions, of part of her population, shed but a sadder light over the comfortless huts of the peasant.* To remedy some of the more pressing evils, Mr. Phelan proposes a scheme of poor-laws. The limits of this article only permit us to present a condensed notice of the actual state of the Irish medical charities.

The condition of the sick poor in the epidemic or endemic fevers produced, and perhaps become hereditary, among these ill-fed, crowded, unhappy people, surpasses conception. In the epidemic of 1827-8-9, scenes like the following were familiar to the medical men in many parts of Ireland.

"In the autumn of 1828," says the medical officer of a dispensary, "fever raged with extreme violence: there being no asylum whatever, temporary huts were erected by the friends of the sick, into many of which whole families were put, consisting of from five to twelve individuals. These huts were composed of the most wretched materials, and the interior (about ten feet by six) beggars all description. Many were lost in these places for want of proper ventilation, and from an accumulation of every kind of filth: comforts we only know by name, even to a drink of whey. The result of my attendance on these squalid abodes of wretchedness was that I took fever, of which I recovered with difficulty." (P. 142.) "Dr. Jacob, of Maryborough, says, 'No idea can be formed of the extent of destitution among the labouring classes. * * While we have unfortunate wretches *literally dying in ditches* when seized with fever or other diseases apprehended to be contagious, we must admit that this country is only in a state of half-civilization.'" (P. 296-7.)

Let us turn from the patients to the institutions for their relief.

It is greatly to be regretted that, where the sources of charity are scanty, they should be inefficiently directed; and in Ireland we fear this has been the case. No public documents exist by which the number or the expenditure of the public medical institutions can be ascertained, although the government may have procured such a return, as the greater part of them receive public aid. Mr. Phelan, however, has collected from Parliamentary Reports and his own enquiries the following account, which he considers an approximation to the truth. (P. 2.)

* "Children are often found dead under suspicious circumstances." (P. 49.) "The custom of supporting their parents is decaying fast." "Being always at home, the daughter-in-law is apt to find her husband's father in the way, and you will see the old man cowering in the corner of the chimney, as if he was endeavouring to hide himself from her." (P. 230.) "The aged labourer is driven to mendicancy, and the gentry never give to beggars. High walls surround their demesnes, and a dog is kept at the gates to prevent the entrance of a beggar." See First Report from his Majesty's Commissioners for Enquiring into the Condition of the Poorer Classes in Ireland. 8th July, 1835.

| | Number. | Expend. |
|---|---|---|
| Dublin Hospitals, (exclusive of several Lying-in Hospitals and Ophthalmic Institutions lately established . . | 7 | £28,701 |
| County and other provincial Infirmaries . . | 38 | 26,426 |
| Fever Hospitals, including three in Dublin . . | 64 | 13,607 |
| Dispensaries . . . . | 528 | 60,000 |
| District Lunatic Asylums . . . . | 11 | 22,965 |
| Total annual expenditure . | 648 | £151,699 |

This does not include £10,000 (probably) expended on medical attendance in workhouses; nor the funds raised by subscription for dispensaries, &c., amounting to not less than £14,000.

The sources whence this expenditure is supplied are:

| | |
|---|---|
| Treasury grants to county infirmaries . . . | £ 2,653 |
| Annual parliamentary grants to the Dublin hospitals . . | 14,374 |
| County presentments . . . . . | 82,839 |
| Subscriptions and donations . . . . | 39,078 |
| Petit sessions and other fines . . . . | 1,742 |
| Produce of property attached to several hospitals . . | 23,225 |
| Total . | £163,911 |

The medical institutions of Dublin admitted about 135,000 patients in 1833; and, as the population of Dublin, and of the country five miles round, is about 315,000, it is inferred that the proportion of persons relieved, compared with the population, is nearly double as great in Dublin as in London. The Dublin hospitals contain about 685 beds, or one bed to 564 persons; the Irish provincial infirmaries contain but 1,262 beds, or one bed for every 5,827 of the population of Ireland, exclusive of Dublin. The total infirmary accommodation in all Ireland is therefore 1,947 beds, which is in the proportion of one bed to every 4000 of the population.

The Dublin hospitals (of the infirmary class) admitted 7,114 in-patients in one year; of these, 4,795 were residents of the city and a circle of about five miles round it; the remaining 2,319 came from beyond that distance; several from the more remote counties. There was, then, one hospital in-patient for every 66 inhabitants, on an average of three years' returns, (1825, 1828, 1832;) all the Irish *county* infirmaries admitted 78,461 in-patients annually, and the others, belonging to cities and towns, 2,104; in all, 9,950 in-patients in one year; a proportion of one to 746 of the total population. The 2,319 received in the Dublin hospitals make a total of 12,269 in-patients relieved annually in the Irish infirmaries; 1-608th of the population. Only 36 out of 266 towns and cities in Ireland, of more than 1000 inhabitants, have infirmaries. Mr. Phelan concludes that Ireland, which is in greater need of hospitals than England, is less adequately supplied.

*Fever Hospitals.* It is stated, in an official work by Dr. Barker and Dr. Cheyne, that, during the epidemic fever which prevailed two years and a half (1816-18) in Ireland, a million and a half, or a fourth of the population, were attacked, and 65,000 died. At this time seventy-two fever hospitals were established in different parts of the kingdom. Mr. Phelan thinks that, in ordinary seasons, 108,000 cases of fever occur annually; 15,000 of which find accommodation in the hospitals.

"Fever," he says, "has been seldom less prevalent than in the three years ending 1831; yet, during these years, the Cork-street and Hardwicke Fever Hospitals in Dublin, and the fever wards of the new Meath Hospital, admitted 5,702 fever patients annually, giving a proportion of one to 66½ of the whole population of the city and county of Dublin, from which alone, with so few exceptions as scarcely to deserve notice, there were sent 61,516."

*Dispensaries* are very numerous, but inefficient: in the county of Down there is only one to a population of 35,000.

The manner in which the infirmaries are supported, managed, and supplied with medical advice in Ireland, deserves examination, and in many cases revision. In England it is well known that all the hospitals, except St. Bartholomew's and St. Thomas's, were founded by private individuals; are principally supported by annual subscriptions, except Guy's; and are under the management of officers chosen by the subscribers: in Ireland, on the contrary, the infirmaries were founded by Act of Parliament, and are supported by treasury or parliamentary grants and county presentments; the subscriptions constituting less than a fourth of the entire revenues.

*History of Infirmaries in Ireland, from Acts of Parliament.* The 5 and 6 Geo. III. (of the Irish Parliaments) made provision for the erection and establishment of one infirmary in each county in Ireland; for the management of which the Primate, the Lord Chancellor, the bishops of the diocess, and the rector or vicar of the parish, with donors of twenty guineas and annual subscribers of three guineas, form a perpetual corporation. Dublin graduates in medicine and surgery are declared to be alone eligible to the offices of physicians and surgeons. The sum of 100*l.* a year is ordered to be paid to each infirmary surgeon by the public treasury; and grand juries are authorized to present from 50*l.* to 100*l.* a year on each county, for the use of its infirmary. The 45 Geo. III. enabled grand juries to present 600*l.* a year on each county for the infirmary, and first made provision for the establishment of dispensaries in Ireland. The 46 Geo. III. provides for the inspection of infirmaries. The 54 Geo. III. enabled grand juries to present an additional sum of 100*l.* a year, to be paid the surgeon of each infirmary; it prohibited all annual subscribers from voting unless two years' subscription had previously been paid, and authorizes governors to establish, if they think proper, a second infirmary in the county; in which case, the salaries of the surgeon (200*l.*) and apothecary (30*l.*) were to be equally divided. Successive Acts of Parliament provided for the establishment of fever hospitals, lunatic asylums, and dispensaries. Only eight or ten houses of industry exist in Ireland, although facilities have been afforded for their establishment. (Pp. 20-32.)

Mr. Phelan candidly admits that the medical officers of the county infirmaries are well qualified for their office, and that these institutions do great service to Ireland; but he maintains that they do not afford *efficient* medical aid to his sick countrymen, and are marred by several defects—not to use a harsher term. These are principally defects in the Acts of Parliament, and in the regulations of the local governors, and may be briefly summed up under the following heads. (P. 42.)

1st. Inconvenience of the sites originally chosen for many of the county infirmaries. They were fixed at the assize towns, which, like Wexford, are sometimes at the outer boundary of the population; and this very much impedes their usefulness. In twelve counties and a half, 4,161 patients entered their respective infirmaries in one year; a population of about 500,000, residing within five miles of the infirmaries, supplied 2,200 of the patients, while the remaining population of 2,600,000 souls furnished but 1,886 patients. The districts contiguous to the hospitals obtained infirmary accommodation for one in 234 of the population; the remoter districts only for one in 1,416.

2d. Vesting the power of establishing a second hospital in each county in the governors of the one first erected. Since this power was given, it has only been exerted once, although much called for.

3d. Giving a monopoly to Irish practitioners *who graduate in Dublin;* whereas, three graduate in Great Britain for one who obtains a degree in Dublin.

4th. The mode of raising the funds, which is inadequate and injurious: for the 700*l.* raised by presentment, and 92*l.* obtained from the treasury, often suffice to carry on the machinery of the institution, and no exertions are made to obtain subscriptions. In 1828, the whole amount of donations, subscriptions, petit-session fines, and other contingencies, in twenty-nine counties, only amounted to 1,973*l.*; on an average, 68*l.* from the nobility, gentry, magistrates, and clergymen of each county. The treasury grants and county presentments were to the amount of 17,022*l.* In King's County, having a population of 144,000, where there are 166 magistrates, about thirty protestant rectors, and thirty catholic priests, besides private gentlemen, there were, on an average of three years, only three life-governors and six annual subscribers. The annual expenditure of the infirmary was 695*l.*

5th. The want of efficient control over institutions supported by public grants: the returns are mockeries.

6th. The annual subscription of three guineas, required for qualifying governors, is too high. Patients cannot obtain recommendations. There are only *two governors* in Galway, although the population amounts to 30,000.

7th. The governors meet very rarely, as they are few, and often absentees.

8th. There being no resident surgeon, and only one medical officer, generally in full practice, attached to each infirmary, the patients are often neglected, and other medical men are excluded.

9th. There is no efficient provision for relieving out-patients at their own houses; which is particularly felt, as there are no parish medical attendants in Ireland.

10th. Surgeons reside at ten hospitals, but in some cases they appear to monopolize, to a very considerable extent, the space which should be allotted to the sick. Mr. Phelan discovered that, in the Kildare Infirmary, which is much too small for the county, the surgeon occupied rooms capable of holding fifteen beds; at Down, the resident surgeon takes up the space of twenty-four beds; and at another place this officer actually has apartments capable of containing forty-eight patients, besides "two stables, two coach-houses, harness-room, barn, and several other offices." He also enjoys his full salary of 192*l.* annually, in the "poor country of Ireland."

11th. The treasurer has too much power at the elections. Up to 1833, subscribers could only vote at the end of two years; but donors of twenty guineas possessed the right of voting immediately on paying their donations. The treasurer alone at an election knows how many pay their donations. Drs. A. and B. are candidates, both willing to purchase the office by making governors. A. gains the treasurer; he ascertains that there are forty votes, and has only a promise of eighteen, which are made a majority by giving the treasurer an order for 120 guineas, to make six donors, as well as power over a sufficient sum to make at all events one more than B. No catholic holds the situation of physician or surgeon to a county infirmary in Ireland.

The elaborate tables at the end of the volume have been compiled with great care, and the materials from which they were deduced must have been collected with great expense. The author not only addressed circulars to the different institutions in Ireland, but corresponded with several gentlemen best acquainted with similar institutions in England. On the whole, the statistical department of Mr. Phelan's work is executed with great ability, and makes us hope that the government will avail themselves of Mr. Phelan's assistance in any system they may propose for the regulation of the Irish medical charities.

The parts of the work referring to the London medical institutions contain several errors; more excusable as, when Mr. Phelan was writing, no accurate information existed on the subject.

